# Diagnostic Ability of a Smartphone App for Dry Eye Disease: Protocol for a Multicenter, Open-Label, Prospective, and Cross-sectional Study

**DOI:** 10.2196/45218

**Published:** 2023-03-13

**Authors:** Ken Nagino, Yuichi Okumura, Masahiro Yamaguchi, Jaemyoung Sung, Masashi Nagao, Kenta Fujio, Yasutsugu Akasaki, Tianxiang Huang, Kunihiko Hirosawa, Masao Iwagami, Akie Midorikawa-Inomata, Keiichi Fujimoto, Atsuko Eguchi, Yukinobu Okajima, Koji Kakisu, Yuto Tei, Takefumi Yamaguchi, Daisuke Tomida, Masaki Fukui, Yukari Yagi-Yaguchi, Yuichi Hori, Jun Shimazaki, Shuko Nojiri, Yuki Morooka, Alan Yee, Maria Miura, Mizu Ohno, Takenori Inomata

**Affiliations:** 1 Department of Hospital Administration Juntendo University Graduate School of Medicine Tokyo Japan; 2 Department of Ophthalmology Juntendo University Graduate School of Medicine Tokyo Japan; 3 Department of Digital Medicine Juntendo University Graduate School of Medicine Tokyo Japan; 4 Morsani College of Medicine University of South Florida Tampa, FL United States; 5 Department of Orthopedics Juntendo University Faculty of Medicine Tokyo Japan; 6 Medical Technology Innovation Center Juntendo University Tokyo Japan; 7 Graduate School of Health and Sports Science Juntendo University Tokyo Japan; 8 Department of Health Services Research, Faculty of Medicine University of Tsukuba Ibaraki Japan; 9 Department of Ophthalmology Toho University Omori Medical Center Tokyo Japan; 10 Department of Ophthalmology Tokyo Dental College Ichikawa General Hospital Chiba Japan; 11 AI Incubation Farm Juntendo University Graduate School of Medicine Tokyo Japan

**Keywords:** digital health, digital therapeutics, dry eye disease, Japanese version of the Ocular Surface Disease Index, maximum blink interval, mobile health, smartphone, smartphone app, tear film breakup time, telemedicine

## Abstract

**Background:**

Dry eye disease (DED) is one of the most common ocular surface diseases. Numerous patients with DED remain undiagnosed and inadequately treated, experiencing various subjective symptoms and a decrease in quality of life and work productivity. A mobile health smartphone app, namely, the DEA01, has been developed as a noninvasive, noncontact, and remote screening device, in the context of an ongoing paradigm shift in the health care system, to facilitate a diagnosis of DED.

**Objective:**

This study aimed to evaluate the capabilities of the DEA01 smartphone app to facilitate a DED diagnosis.

**Methods:**

In this multicenter, open-label, prospective, and cross-sectional study, the *test method* will involve using the DEA01 smartphone app to collect and evaluate DED symptoms, based on the Japanese version of the Ocular Surface Disease Index (J-OSDI), and to measure the maximum blink interval (MBI). The *standard method* will then involve a paper-based J-OSDI evaluation of subjective symptoms of DED and tear film breakup time (TFBUT) measurement in an in-person encounter. We will allocate 220 patients to DED and non-DED groups, based on the *standard method*. The primary outcome will be the sensitivity and specificity of the DED diagnosis according to the *test method*. Secondary outcomes will be the validity and reliability of the *test method*. The concordance rate, positive and negative predictive values, and the likelihood ratio between the *test* and *standard methods* will be assessed. The area under the curve of the *test method* will be evaluated using a receiver operating characteristic curve. The internal consistency of the app-based J-OSDI and the correlation between the app-based J-OSDI and paper-based J-OSDI will be assessed. A DED diagnosis cutoff value for the app-based MBI will be determined using a receiver operating characteristic curve. The app-based MBI will be assessed to determine a correlation between a slit lamp–based MBI and TFBUT. Adverse events and DEA01 failure data will be collected. Operability and usability will be assessed using a 5-point Likert scale questionnaire.

**Results:**

Patient enrollment will start in February 2023 and end in July 2023. The findings will be analyzed in August 2023, and the results will be reported from March 2024 onward.

**Conclusions:**

This study may have implications in identifying a noninvasive, noncontact route to facilitate a diagnosis of DED. The DEA01 may enable a comprehensive diagnostic evaluation within a telemedicine setting and facilitate early intervention for undiagnosed patients with DED confronting health care access barriers.

**Trial Registration:**

Japan Registry of Clinical Trials jRCTs032220524; https://jrct.niph.go.jp/latest-detail/jRCTs032220524

**International Registered Report Identifier (IRRID):**

PRR1-10.2196/45218

## Introduction

Dry eye disease (DED) is one of the most frequently encountered ocular surface diseases, with a prevalence ranging from 5% to 50% [1,2], which is projected to increase, likely due to the worldwide trend toward an aging population and lifestyle changes associated with a digitalized society [1-4]. DED-related symptoms are detrimental to quality of life (QoL) and work productivity, leading to society-wide economic loss [5,6]. Subjective DED-related symptoms are highly variable and include ocular dryness, eye discomfort, eye strain, decreased visual acuity, and photophobia [7-11]. Additionally, DED is highly heterogeneous, with patients presenting with seemingly unpredictable combinations of the abovementioned symptoms. Therefore, patients with DED are prone to having their nonspecific presentations overlooked, and many are left undiagnosed without appropriate management [3,9,12-14]. There is no definitive cure for DED, and current standards of care comprise ex post facto symptom management, highlighting the importance of early diagnosis and intervention to prevent disease progression and longstanding damage [15-19].

Concerning DED diagnosis, Tear Film & Ocular Surface Society and Asian Dry Eye Society criteria recommend evaluating a patient’s subjective symptoms and tear film breakup time (TFBUT) [20,21]. However, TFBUT measurement is inherently invasive owing to the need for sodium fluorescein application, and measurements under light microscopy are required [20-23]. The ongoing COVID-19 pandemic has also increased the demand for a noninvasive alternative that could be used in remote settings [24,25]. One major obstacle to DED diagnosis in the context of telemedicine is the inability to evaluate the ocular surface and the use of slit-lamp microscopy in TFBUT, and clinicians may be solely reliant on subjective symptoms for diagnosis. Therefore, identification of a novel proxy for existing invasive examinations is needed for a reliable, noninvasive DED diagnosis.

Recent studies have suggested the maximum blink interval (MBI) measurement as a noninvasive alternative to TFBUT, with the MBI defined as the maximum length of time a patient is able to keep their eyes open between 2 blinks [26]. A positive correlation has been reported between the MBI and TFBUT, likely owing to the MBI’s capability to reflect ocular surface integrity [26-30]. The simplicity and noninvasive nature of MBI measurements underscores its candidacy as an alternative to TFBUT for screening purposes. Our earlier studies have indicated that the concomitant use of the MBI and the Ocular Surface Disease Index (OSDI) questionnaire can determine DED with similar accuracy to standard diagnostic methods using TFBUT and the OSDI [31,32].

Mobile health (mHealth) is defined by the World Health Organization as “medical and public health practice supported by mobile devices, such as mobile phones, patient monitoring devices, personal digital assistants (PDAs), and other wireless devices” [33]. The capability of mHealth to digitally perform therapeutic activities has raised the interest of clinicians and providers due to its potential use in disease prevention, diagnosis, and treatment through commonplace smart devices [32,34-39]. Previous studies concerning the validity and reliability of smartphone-based administration of MBI examinations and OSDI questionnaires yielded a satisfactory result [3,32,40]. Reported findings suggest that noninvasive and nonintrusive DED screening during activities of daily living may be possible, enabling an earlier DED diagnosis and interventions in remote settings [40,41]. More importantly, this possibility may have implications in accurately identifying undiagnosed patients with DED, allowing for timely intervention, preventing a further decrease in QoL, and reducing societal economic loss.

In this clinical study, we intend to use an in-house smartphone app, namely, the DEA01, which has been designed as an investigational device to facilitate a DED diagnosis. This study will aim to evaluate the capabilities of the DEA01 in facilitating a diagnosis of DED.

## Methods

### The DEA01 Smartphone App

The DEA01 smartphone app for DED diagnostic assistance was developed by InnoJin, Inc. It will be available on Apple’s App Store and the Google Play Store. As of December 2022, it has not yet been approved as software for a medical device in Japan.

**Figure 1:**
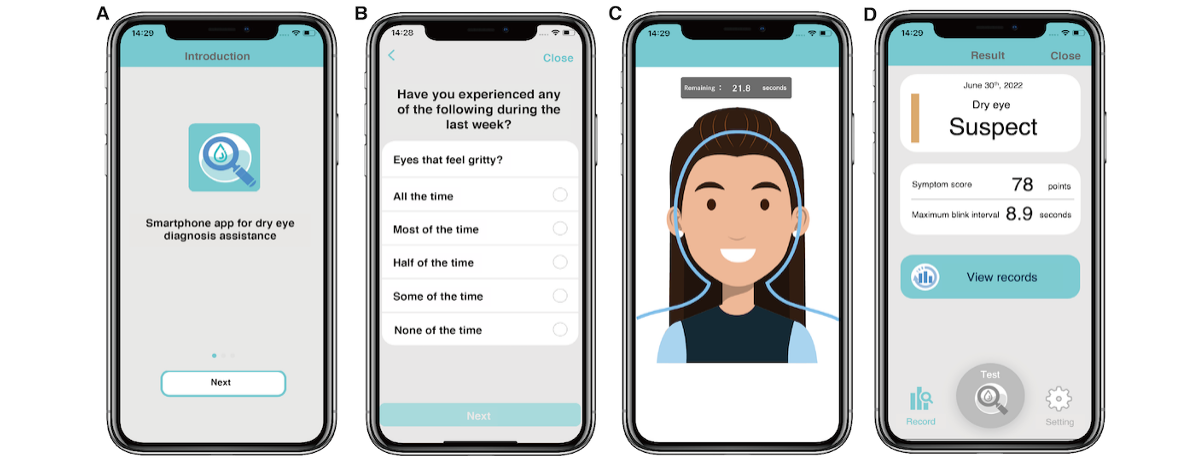
Screenshots of DEA01: screenshots of the (A) welcome screen, (B) Ocular Surface Disease Index (OSDI) questionnaire, (C) measurement of the maximum blink interval, and (D) examination result screen for dry eye diagnostic assistance.

The DEA01 collects dry eye symptoms using the OSDI and MBI to facilitate a DED diagnosis [32]. Blinking is measured using the smartphone camera and the *CIFaceFeature* function in the iOS interface for facial detection. DEA01 version 1.0 will be installed on an iPhone 13 Pro (Apple Inc). The app will be modified should minor issues arise during the study. Screenshots of the current version of the DEA01 (December 2022) are shown Figure 1.

### Objective

This study’s primary objective is to evaluate DED diagnostic accuracy using the DEA01 smartphone app compared with standard DED diagnostic methods.

### Study Design

**Figure 2:**
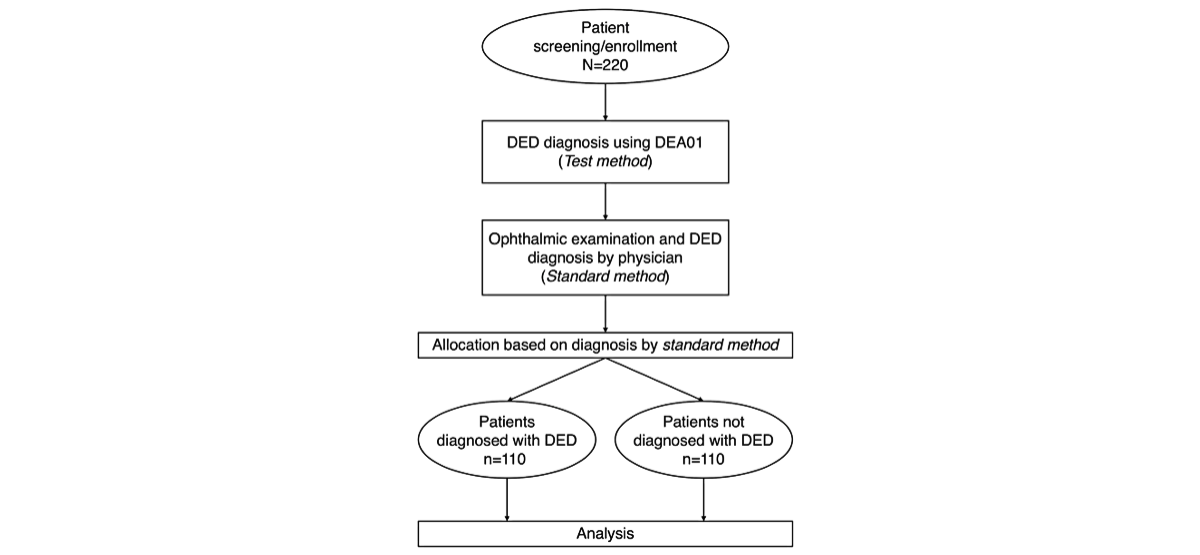
Study flowchart. DED: dry eye disease.

In this multicenter, open-label, prospective, and cross-sectional study, patients will be evaluated for DED using the DEA01 (*test method*). A physician will then provide a DED diagnosis based on 2016 Asia Dry Eye Society criteria [20] (*standard method*). To ensure that the *test method* will not affect a patient’s treatment plan, the physician will make a diagnosis using only *standard method* results without reviewing *test method* results. A minimum number of 220 patients will be enrolled, with patients allocated into a DED group or a non-DED group based on the diagnosis obtained using the *standard method*. The study design is shown in Figure 2.

### Ethics Approval

This study has been approved by the Juntendo University Certified Review Board, Tokyo, Japan (approved protocol V.1.1.1, dated December 19, 2022; approval number: J22-003). Patients will have the option to voluntarily provide written informed consent to participate in the study. Written consent will be obtained from a patient’s parent or legal guardian for patients aged <20 years. All involved parties will make every effort to protect study patients’ personal information and privacy. Patient-related data will be anonymized, and research-related data and the smartphones used in this study will be stored in locked lockers at each collaborating institution, with access strictly controlled by the research staff. All patients will receive a 1000-yen (US $7.77) voucher.

### Study Setting

This study will be conducted between February 1, 2023, and November 30, 2023. Patients will be recruited from the Departments of Ophthalmology at 3 medical institutions, namely, Juntendo University Hospital, Tokyo, Japan [42]; Toho University Omori Medical Center, Tokyo, Japan; and Tokyo Dental College Ichikawa General Hospital, Chiba, Japan.

### Patient Selection

Outpatients who visit the Ophthalmology Departments of the cooperating hospitals will be recruited for the study. Enrolled patients will include those with DED and other concomitant ocular diseases. Inclusion and exclusion criteria are shown in Textbox 1.

**Inclusion and exclusion criteria.**

**Inclusion criteria**

1. Patients with dry eye disease (DED) or suspected DED
2. Patients aged ≥16 years at the time of providing informed consent
3. Patients able to use smartphones
4. Patients/guardians who, after receiving a full explanation concerning participation in the study and with a full understanding of the study, voluntarily provide written consent to participate

**Exclusion criteria**

1. Patients with eyelid closure disorder, ptosis, psychiatric disorders, and Parkinson disease
2. Patients who wear contact lenses
3. Patients who are unable to comply with the study protocol for physical or mental reasons
4. Patients whom the principal investigator deems unsuitable to participate in this study

### Patient Withdrawal

Patients will be withdrawn from the study if, due to the occurrence of other diseases, it is deemed difficult to continue the research; if patients or their parents/guardians request to terminate their participation in the research; if the research study is discontinued; or if the principal investigator and subinvestigators determine that it is appropriate to discontinue the research.

### Study Procedures

After enrollment in the study, patients will be evaluated for DED by clinical research coordinators using the DEA01. A physician will diagnose DED using the *standard method* without checking *test method* results. Details regarding the *test method* and *standard method* are shown in Textbox 2.

***Test* and *standard method.***

***Test method***

- Dry eye disease (DED) diagnosis based on the app-based Japanese version of the Ocular Surface Disease Index (J-OSDI) and maximum blink interval (MBI), using the DEA01
- Patients will respond to the app-based J-OSDI questionnaire and be measured using the app-based MBI on the DEA01. The J-OSDI questionnaire is used to evaluate the subjective symptoms of DED on a 100-point scale [43,44]. The MBI will be defined as the length of time that a patient can keep their eyes open before blinking [26]. The app-based MBI will be measured using the smartphone’s camera on the DEA01 and a visually confirmed MBI will be measured using a stopwatch.

***Standard method***

- DED diagnosis using a paper-based J-OSDI and tear film breakup time (TFBUT)
- Patients will respond to the paper-based J-OSDI questionnaire and be measured using TFBUT [20]. The diagnosis of DED using the *standard method* will be based on 2016 Asian Dry Eye Society criteria, with positive subjective symptoms (J-OSDI total score ≥13) and decreased TFBUT (≤5.0 s) being used to diagnose DED [20]. Based on the diagnosis using the *standard method*, patients will be allocated to DED and non-DED groups.

### Data Collection and Outcome Assessments

The data collection items are shown in Textbox 3. The assessments will be done according to a predetermined sequence. First, the assessment of subjective symptoms of DED using the app-based J-OSDI and MBI on the DEA01 will be conducted as the *test method* by trained clinical research coordinators. The physician will not be informed of the *test method* results. The physician will then perform clinical examinations for DED, using the *standard method* and ophthalmic examinations. Clinical examinations for DED will include an evaluation of subjective symptoms of DED using the paper-based J-OSDI questionnaire, slit lamp–based MBI, TFBUT, corneal fluorescein staining (CFS), and tear secretion volume using Schirmer test I. The ophthalmic examinations will include measurement of visual acuity, intraocular pressure, and slit-lamp microscopic photography. All examinations will be performed within one day.

**Data collection.**

**Patients’ characteristics**

- Age, sex, medical history, and medication

***Test method***

- Subjective symptoms of dry eye disease (DED) evaluated using the app-based the Japanese version of the Ocular Surface Disease Index (J-OSDI) on the DEA01
- Visually confirmed maximum blink interval (MBI)
- App-based MBI

***Standard method***

- Subjective symptoms of DED evaluated using the paper-based J-OSDI
- Tear film breakup time

**Other DED examinations**

- Corneal fluorescein staining
- Slit lamp–based MBI
- Tear secretion volume using Schirmer test I

**Ophthalmic examinations**

- Visual acuity
- Intraocular pressure
- Slit-lamp microscopy

**Other outcomes**

- DEA01 failure
- DEA01 operability and usability
- Adverse events

#### The Japanese Version of the OSDI

The Japanese version of the OSDI (J-OSDI) is a questionnaire comprising 12 questions to evaluate subjective symptoms of DED and has been validated in Japan as an OSDI-equivalent questionnaire for DED diagnosis [43-45]. Patients will answer each question on a scale ranging from 0 to 4, with 0 indicating “none of the time” and 4 indicating “all of the time.” For questions 6 through 12, if a question is not applicable to a patient, they can select “not applicable.” The J-OSDI results are reported on a 100-point scale to determine the severity of DED symptoms. The J-OSDI total score is calculated according to the following formula [43,44]:



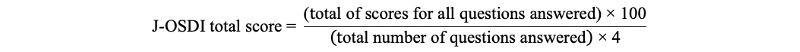

**(1)**


#### TFBUT

TFBUT will be measured using sodium fluorescein to assess tear film stability [20]. To minimize the effect of a test strip on tear volume and TFBUT, a small amount of dye will be administered using wet fluorescein strips. After the dye is instilled, the patients will be instructed to blink 3 times to ensure that the dye is uniformly incorporated into the tear film. The time interval between the last blink and the appearance of the first dark spot on the cornea will be measured, and the average value from 3 measurements will be used as the test outcome. A cutoff TFBUT value of ≤5.0 seconds will be used to diagnose DED [20].

#### MBI

The MBI is defined as the length of time that a patient is able to keep their eyes open before blinking during each trial [26]. In the *test method*, the app-based MBI will be measured using the smartphone’s camera on the DEA01, and visually confirmed MBI will be measured using a stopwatch by a trained clinical research coordinator. In the *standard method*, the MBI will be measured by a physician using a slit-lamp microscope. In both methods, the MBI will be measured twice for each patient and the average value will be adopted as the result. An MBI exceeding 30 seconds will be recorded as 30 seconds. The MBI cutoff value will be calculated using receiver operating characteristic (ROC) curve analysis [31].

#### CFS

CFS will be assessed under a slit-lamp microscope with blue light and a yellow band–pass filer according to the van Bijsterveld grading system, in which the ocular surface will be divided into 3 zones: the nasal bulbar conjunctiva, temporal bulbar conjunctiva, and cornea [46]. Each zone will be evaluated on a scale from 0 to 3, with 0 indicating no staining and 3 indicating confluent staining. The maximum potential score is 9.

#### Schirmer Test I for Tear Secretion Volume

Schirmer test I will be performed without topical anesthesia after all other examinations. A Schirmer test strip will be placed on the outer one-third of the temporal lower conjunctival fornix for 5 minutes. The length of the moisture stain on the filter paper (in mm) will be recorded [47].

#### Environmental Conditions

The temperature of the examination room will be set at 26 °C in summer and at 24 °C in winter, with a constant relative humidity of 50%, following *Guidelines for Design and Operation of Hospital HVAC Systems*, established by the Healthcare Engineering Association of Japan [48].

### Primary Outcome

The primary outcome will be the sensitivity and specificity of the *test method* for DED diagnosis, compared with the *standard method*. Sensitivity and specificity will be calculated based on the diagnostic results of the 2 methods. In the *test method*, an app-based J-OSDI total score ≥13 measured using the DEA01 and an MBI score greater than the cutoff value based on the secondary outcome (5) will be considered DED positive. In the *standard method*, a paper-based J-OSDI total score ≥13 and TFBUT ≤5.0 seconds will be considered DED positive.

### Secondary Outcomes

Secondary outcomes are as follows:

1. An overall concordance rate between the *test method* and *standard method*
2. A positive/negative predictive value between the *test method* and *standard method*
3. A positive/negative likelihood ratio between the *test method* and *standard method*
4. An area under the ROC curve of the *test method* for DED diagnosis using the *standard method*
5. An MBI cutoff value of the *test method* for DED diagnosis using the *standard method*
6. An area under the ROC curve of the *test method* for TFBUT ≤5.0 seconds
7. An MBI cutoff value of the *test method* for TFBUT ≤5.0 seconds
8. Internal consistency of the app-based J-OSDI
9. Correlation, agreement, and comparison between the app-based J-OSDI and paper- based J-OSDI
10. Correlation, agreement, and comparison between the app-based MBI and slit lamp– based MBI
11. Correlation and comparison between TFBUT and the app-based MBI
12. Correlation and comparison between TFBUT and slit lamp–based MBI
13. DEA01 operability and usability
14. DEA01 failure
15. Adverse events

Secondary outcomes 1 to 4 were set to evaluate the DED diagnosis accuracy of the *test method* compared with that of the *standard method*. Secondary outcome 5 was included to determine the cutoff value for the app-based MBI using ROC analysis for DED diagnosis. Secondary outcomes 6 and 7 were included to determine if MBI measurements could be used as a substitute for TFBUT measurements. Secondary outcomes 8 and 9 were set to evaluate the reliability and validity of the app-based J-OSDI. Secondary outcomes 10 to 12 were set to evaluate the validity of app-based and slit lamp–based MBIs. In secondary outcome 13, patients will answer the following questions: “I think it’s easy to operate this smartphone app,” “I can use this smartphone app by myself,” “I want to be tested using this smartphone app rather than being tested at a medical institution,” and “I will continue to use this smartphone app” [49,50]. Patients will answer these questions on a 5-point Likert scale (1, strongly disagree; 2, disagree; 3, neutral; 4, agree; and 5, strongly agree) [51]. Secondary outcomes 14 and 15 were set to evaluate the safety and stability of the DEA01. DEA01 failure is defined as something not working as expected. Adverse events comprise unexpected signs, symptoms, or diseases encountered during the clinical trial, regardless of whether they are related to treatment. If serious adverse events or failure occur, these will be referred to the Juntendo Hospital Certified Review Board, and appropriate treatments will be provided as needed.

### Sample Size and Statistical Analyses

In total, the enrollment of a minimum number of 220 study patients will be required. The CI for the sensitivity and specificity of the *test method* is set at ±10%. It is possible to calculate a sample size and a CI (a margin of error) using the following formula [52]:



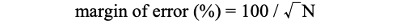

**(2)**


Therefore, the minimum sample size will be 100 patients in each group. To meet the minimum required number of patients in each group, the target total number of patients will be set at 110 for each group, assuming a 10% exclusion rate due to missing data.

For the primary end points, the sensitivity and specificity of DED diagnosis using the DEA01 will be calculated using ROC curve analysis with the cutoff value for the app-based MBI in the *test method*. This cutoff value will be calculated using ROC curve analysis [31]. The ROC curve will show sensitivity (positive rate) on the vertical axis and 1-specificity (false positive rate) on the horizontal axis, and the plots will be connected using straight lines. The point at which the sensitivity and specificity for DED diagnosis using the app-based MBI will be maximized and will be set as the cutoff value for MBI [31].

The secondary outcomes of the overall concordance rate, positive/negative predictive value, and positive/negative likelihood ratio will be calculated by comparing the DED diagnosis results from the *test method* with the results from *standard method* in a mixed matrix. The diagnostic performance of DEA01 will be evaluated through calculating the area under the ROC curve plotted from the sensitivity and specificity of the *test method*. The substitutability of MBI measurement for TFBUT measurement in DED diagnosis will be assessed using ROC curve analysis and through calculating the area under the ROC curve, using the cutoff value of the app-based MBI for TFBUT ≤5 seconds. The internal consistency of the app-based J-OSDI will be evaluated using factor analysis and Cronbach α coefficient, with α>0.70 considered an acceptable range [53]. Correlation and consistency will be evaluated using Pearson or Spearman correlation coefficients, an intraclass correlation coefficient, and Bland-Altman analysis. In each patient group, study patients’ backgrounds will be analyzed using a 2-tailed unpaired *t* test for continuous variables and a chi-square test for categorical variables. If a continuous variable does not clearly follow a normal distribution, it will be appropriately transformed to a logarithm and aggregated with the mean and SD or the median and IQR will be used as descriptive statistics. The operability and usability of the DEA01 will be evaluated in each group using the average score of the 5-point Likert scale responses to the operability and usability questions and the number of patients who answered better or worse than neutral using 2-tailed unpaired *t* and chi-square tests. For safety assessment, the frequency and rate of adverse events will be calculated for each group, and between-group comparisons will be performed using Fisher exact probability or chi-square tests.

A 2-sided level of significance will be set at 5%, and the confidence coefficient will be set at 95%, unless otherwise specified. Missing value completion will not be performed.

### Data Management

Data management will be conducted at an off-campus data center designated by the Juntendo University Certified Review Board in accordance with a preprepared data management plan. Data will be collected and linked at several points in time. Following database locking, locked data will be transferred to the person responsible for statistical analysis.

## Results

This study will start on February 1, 2023, at 3 medical institutions in Japan. Patient enrollment is expected to begin on February 1, 2023, and will end on July 31, 2023. Data will be analyzed in August 2023, and the results will be reported from December 2023 onward.

## Discussion

A recent report suggested that a significant portion of patients with DED might currently be left undiagnosed and experiencing a preventable decrease in QoL [13]. For those who are appropriately diagnosed, barriers to receiving care remain, including limitations related to employment, education, and the ongoing COVID-19 pandemic. Hence, there is a need for a nonintrusive, telehealth-based alternative to current in-person evaluations. In this study, the capabilities of a smartphone app-based diagnostic assistive device will be investigated in a multicenter, open-label, prospective, and cross-sectional study. The results are intended to demonstrate the reliability and validity of the DEA01 as a noninvasive tool to facilitate a diagnosis of DED, through determining the appropriate MBI cutoff value. Confirmation of the value of the DEA01 is likely to facilitate early intervention and greater outreach to undiagnosed populations with DED.

The health care paradigm is rapidly shifting to promote remote and online care given societal changes implemented during the ongoing COVID-19 pandemic [32]. Notably, for glaucoma- and diabetes-related retinopathy management, a new approach has been sought to monitor disease progression at home through the use of portable devices and to offer visits for ocular anomalies of concern [54]. This study aims to establish a new means to facilitate DED diagnosis in a nonintrusive, noninvasive manner using the DEA01 smartphone app, which can yield an objective metric for clinicians to evaluate remotely. This may lead to a global shift toward endorsing telehealth and thus, increasing accessibility and helping to remove barriers to health care. The potential to promote early diagnosis and intervention can dramatically affect disease prognosis on a global scale. Additionally, such apps are well aligned with the current paradigm shift away from standard facility-oriented care and toward a daily life–oriented longitudinal, human-centered health care approach [40,55,56].

The DEA01 is used to help assess subjective symptoms and collect objective findings through OSDI administration and MBI measurements, respectively. The reliability and validity of the MBI and OSDI in a remote setting to facilitate DED diagnosis have been previously shown through a previous DEA01 app designed for DED research, namely, DryEyeRhythm [31,32]. Additionally, discrepancies between results obtained from the standard paper-based OSDI and app-based OSDI have been previously shown to be acceptable [32]. Our prior study utilized the MBI cutoff derived from a slit-lamp microscopy–based MBI study [26]. This study aims to establish an MBI cutoff for smartphone app-based diagnosis assistance for DED. The establishment of an optimal MBI cutoff value will enable more robust and reliable app-based remote DED diagnosis assistance through the use of the DEA01.

In this study, the diagnostic performance utilizing the app-based MBI as a proxy for the standard TFBUT will be evaluated using the DEA01. TFBUT reflects tear film stability and homeostasis, acting as a critical component of DED diagnosis. However, it requires specialized tools and materials, including fluorescein dye and slit-lamp microscopy. Moreover, the invasive nature of the examination disrupts the in vivo status of the tear film and the ocular surface [20-23]. Some studies have shown that fluorescein dye results are not consistently reproducible and that the dye may negatively affect the stability of the tear film [57-59]. The MBI has been shown to have a positive correlation with TFBUT [26], suggesting that the MBI can be used as a noninvasive, nonintrusive metric to substitute for TFBUT. An online screening test, based on a patient’s self-measurement of the time from stopping blinking to feeling discomfort, has shown the utility of screening for DED detection [26,60]. However, the DEA01 may be more objective as it automatically measures the MBI using the sensors of a smartphone camera and allows for the evaluation of DED symptoms using the J-OSDI questionnaire. This study seeks to assess the viability of replacing TFBUT with app-based MBI measurements, which may enable remote, noninvasive, and nonintrusive diagnosis assistance concerning DED.

This study may have some limitations. First, this study may be affected by selection bias, as study patients are limited to those who have been evaluated in a highly specialized university hospital and those who are able to use the smartphone app [32]. Second, the study will be performed in a specific order. Patients will be required to first use the app, after which there will be a clinical evaluation for DED. This study sequence may be a confounder and might affect the results; therefore, assessing the effects of the examination order in a randomized study may be necessary [61]. Lastly, the DEA01 is designed to be an ancillary tool for initial DED diagnosis assistance, and specialized tests such as corneal staining and Schirmer test may be required to further assess disease severity and progression.

In conclusion, this study will evaluate the capability of the DEA01 in assisting a DED diagnosis. The study findings may potentially help to establish a noncontact, noninvasive, and remote consultation method for app-based DED diagnostic assistance using the mHealth DEA01 smartphone app. It is hoped that the DEA01 will provide effective diagnostic evaluation for DED in telemedicine and allow for early intervention in patients who have not been diagnosed with DED due to limited access to medical care.

## Abbreviations

CFS: corneal fluorescein staining
DED: dry eye disease
J-OSDI: Japanese version of the Ocular Surface Disease Index
MBI: maximum blink interval
mHealth: mobile health
OSDI: Ocular Surface Disease Index
ROC: receiver operating characteristic
TFBUT: tear film breakup time
QoL: quality of life
